# Biophotonic Tools in Cell and Tissue Diagnostics

**DOI:** 10.6028/jres.112.011

**Published:** 2007-06-01

**Authors:** Michael Brownstein, Robert A. Hoffman, Richard Levenson, Thomas E. Milner, M. L. Dowell, P. A. Williams, G. S. White, A. K. Gaigalas, J. C. Hwang

**Affiliations:** The J. Craig Venter Institute; BD Biosciences; Cambridge Research and Instrumentation; The University of Texas at Austin; National Institute of Standards and Technology, Boulder, CO 80305-3328

**Keywords:** biophotonics, flow cytome-try, imaging, microarray, optical coherence tomography

## Abstract

In order to maintain the rapid advance of biophotonics in the U.S. and enhance our competitiveness worldwide, key measurement tools must be in place. As part of a wide-reaching effort to improve the U.S. technology base, the National Institute of Standards and Technology sponsored a workshop titled “Biophotonic tools for cell and tissue diagnostics.” The workshop focused on diagnostic techniques involving the interaction between biological systems and photons. Through invited presentations by industry representatives and panel discussion, near- and far-term measurement needs were evaluated. As a result of this workshop, this document has been prepared on the measurement tools needed for biophotonic cell and tissue diagnostics. This will become a part of the larger measurement road-mapping effort to be presented to the Nation as an assessment of the U.S. Measurement System. The information will be used to highlight measurement needs to the community and to facilitate solutions.

## 1. Introduction

Since the invention of the microscope over 300 years ago, light has been used to probe biological samples. With the appearance of laser sources, versatile detectors (e.g., photomultipliers and CCD arrays), and optical filters, the use of light in biological and medical research has become increasingly sophisticated. The interaction between light and biological system leads to the modification of both; unraveling and understanding the changes is the purview of biophotonics [1]. The scope of biophotonic applications can be gleaned from the large number of examples described in recent books edited by Marriott and Parker [2,3].

To discuss diagnostic tools it is useful to have a clear picture of what is being measured. On the most fundamental level, each cell has a fixed content of deoxyribonucleic acid (DNA) (genome) and a certain content of proteins (proteome). As currently understood, most functions of the cell are reflected in the genes that are activated, the amount of proteins expressed, and post transcription modifications that occur. Thus the meaningful measurements for elucidating the detailed state of a cell are the number and type of genes being expressed, and the proteins that are present in the cell. Normal cells are associated with certain characteristic levels and patterns of gene transcription and certain characteristic levels of proteins. Disease states are associated with deviations from these “normal” levels and patterns. The measurement technologies which attempt to give a detailed picture of the genome and proteome are based on microarrays for DNA and proteins. With the development of microarrays there is an expectation that more detailed knowledge of gene expression and protein content can be obtained for diagnostic purposes. For example, patterns of gene expression arrays are useful in differentiating myeloid from lymphoid leukemia. They are even more useful in the classification of heterogeneous lymphoid neoplasmas that cannot be resolved with conventional morphology analysis.

Much of current biological research is aimed at obtaining a detailed understanding of the various pathways in the cell that are associated with normal functions. This research often identifies characteristic genes and proteins associated with a particular function of the cell. Consequently, it also becomes possible to identify genes and proteins associated with malfunctions. These genes and proteins then can serve as “surrogate markers” for a pathological condition at an early, preclinical stage in the development of diseases. The measurement of such biomarkers constitutes a diagnostic assay. A classical example is the measurement of the absolute number of CD4 T cells, CD8 T cells and CD20 B cells based on cell surface antigens in whole blood. The decrease of the CD4/CD8 ratio is associated with HIV disease and an increase in CD20 positive B cells is observed in occurrences of lymphoid leukemia. These measurements are performed on single lymph cells using flow cytometers.

Once a pathological condition has established itself in the body, larger scale (tissue level) abnormalities appear. Traditionally, the detection of deviations in tissue biochemistry has been performed in histology labs, which use microscopes to look at cell morphology and histopathology in biopsy samples. Thus it is possible to detect the presence of tumors, deterioration of artery walls, abnormal growth in neuron sheaths, and other examples. Improvements in image acquisition and analysis have resulted in significant increase in the accuracy of disease detection using the traditional techniques.

The development of optical coherence tomography shows promise for improvement in the detection of subtle morphological changes in vivo [4], and thus speed the detection of disease conditions. Similar expectations have fueled the development of novel *in situ* detection techniques, based on measurement of intrinsic spectroscopic properties in skin and surface layers of tissue such as fluorescence lifetime imaging, multiphoton imaging, hyperspectral imaging, and single molecule detection. Many of the new techniques are in clinical testing and are expected to be used in the future.

The remaining sections of this paper highlight the major measurement challenges that currently limit the usefulness of four common diagnostic techniques involving the interaction of light and biological systems. These four techniques are: Microarrays, Flow Cytometry, Optical Microscopy, and Optical Coherence Tomography. In each section, there are suggested approaches to overcoming these obstacles.

## 2. Measurement Tools and Challenges

### 2.1 Microarrays

The diversity of test sequences and well behaved target/probe interactions of genes allow the design of fully informative assays, based on microarray techniques. Consequently, microarrays have been widely employed as tools to study the role of rare genes (DNA and ribonucleic acid (RNA)) in human diseases, infectious pathogens, and microbes. However, the dearth of microarrays that have been approved by the Food and Drug Administration (FDA) has hindered the use of microarrays as clinical diagnostic tools. The microarray technology is capable of examining thousands of different genes from a single small tissue sample to determine genomic characteristics, for instance, to differentiate between mutated and normal genes. Diagnostic microarrays often contain synthesized DNA sequences on the grid points of a glass slide. The sequences are then hybridized with genomic DNA from the individual patient to determine to which allelic sequences it binds, and therefore which alleles are present in the patient’s genome. An excellent overview of the microarray technology is provided in a recent report [5].

Reliable detection of single pathogenic species and rare transcripts are becoming feasible. Further enhancement of microarray techniques will allow us to look at many targets in parallel, for rapid diagnosis and effective response to infectious diseases, naturally occurring epidemics, and bioterrorist attacks. For instance, the containment of water- and food-borne infections is possible by effective identification of the causes of the infections. In battling against rapidly spreading infectious diseases, the misuse of antibiotics and the lack of incentives to develop new antibiotic and antiviral agents have increased the risk of more serious infections. The ability to differentiate the origins and types of infections will enable rational and highly targeted drug therapy.

Whether the analyte is microbial or human genomic DNA, the objective is to reliably detect as few as one and as many as 100,000 specific DNA or RNA molecules in biological samples. The ideal assay would be fast (real time), specific, sensitive, quantitative, flexible, able to detect many genes in parallel, portable and robust, and inexpensive. Among other gene detection methods, many Polymerase Chain Reaction (PCR)-related techniques have been gradually adopted by clinical laboratories, while array-based assays are still lagging behind because of the hurdles associated with gaining FDA approval. Currently, there are only a few FDA-approved microarray-based assays. One example is a technique for determining single nucleotide polymorphisms in drug metabolizing (P450) enzymes. Some of the factors contributing to the limited number of FDA-approved of microarray-based assays are listed below.

#### 2.1.1 The Identification of Rare Genes From Clinical and Environmental Isolates to Define Clinically Relevant Templates for Microarrays

##### Problem

To detect rare genes and pathogens (ultimately at the single copy level), the sensitivity and accuracy of the detection needs to be improved to overcome current limitations. One of the major hurdles is undesirable optical properties of fluorescent probes, such as monoreactive dyes, which place limits on the sensitivity and quantitation. Their relatively limited dynamic range and unstable fluorescence emission cause serious inaccuracy in parallel assays to detect multiple genes and pathogens on one microarray template.

##### Approach

In addition to the development of optical standards involving conventional organic fluorescence probes, other standards employing novel fluorescent probes have also been proposed and are under development to enhance sensitivity, accuracy, and speed of the technique. Fluorescent nanocrystals, nanoshells, and nanotubes have been identified to have unique optical and physical properties that overcome the limitations of conventional fluorescent probes. For instance, semiconductor fluorescent nanocrystals (NCs) and quantum dots (QDs) exhibit long fluorescence lifetimes, narrow emission spectra, and are not susceptible to photodegradation. QDs not only exhibit higher fluorescence quantum yields than organic dyes, but their longer fluorescence lifetime allows temporal “gating” with respect to background autofluorescence, which enhances the contrast in biological imaging and sensing applications. Only a few nanometers in diameter, QDs are usually composed of a semiconductor core (e.g., CdSe, InP, ZnSe, etc.) capped with a ZnS shell. QDs fluoresce with very broad absorption and narrow emission spectra. By adjusting the core size, the emission wavelength can be finely tuned. The ZnS shell helps in stabilizing the core, makes the light emission more intense, and keeps the QD from degrading. For biological application, it has been demonstrated that the surfaces of NCs can be conjugated with functional groups and molecules, so they can be readily linked to specific DNA sequences for use in microarray platforms. The excellent photochemical and physical properties of QDs and NCs would be excellent surrogates to organic dyes, enabling highly sensitive and quantitative measurements in microarray techniques.

#### 2.1.2 Dearth of Validation Standards for Clinical Applications

##### Problem

For the validation of screening methods for clinical applications of this technique, standards to correlate between clinical diagnostics and screening results need to be established. There is no standard infrastructure or protocol to establish and validate clinically relevant genomic templates for the detection of rare genes. In addition, no validation standards of screening methods for such clinical samples exist. Rapid validation of screening methods and templates to establish clinically applicable assays is of urgent need. According to a recent FDA workshop on microarray techniques [6], FDA’s Critical Path Initiative [7] identifies pharmacogenomics as a key opportunity in advancing medical product development and personalized medicine. The FDA issued the “*Guidance for Industry: Pharmacogenomic Data Submissions*” [8] to facilitate scientific progress in the field of pharmacogenomics and to facilitate the use of pharmacogenomic data in drug development and medical diagnostics. In this guidance, it is identified that exploratory genomic data obtained with microarrays represent one of the core competences in pharmacogenomics; however, this workshop also identified that quantitative flow cytometry applications will be limited until inter-laboratory variability can be controlled and standards are identified [9].

##### Approach

To accelerate this technique towards broader applications including clinical diagnostics, validation techniques in screening gene chip templates for clinical applications and standards to correlate between clinical diagnostics and screening results are needed. To this end, a set of techniques to enable high throughput and high accuracy measurements in relevant fields such as optical characterization of conventional and novel probes and quantitative assessment of clinically relevant gene chip templates needs to be identified and established. A strong research partnership among clinical institutes, government agencies, and other research parties will be central for this purpose. This multi-agency partnership may also solve other challenges including high instrumentation cost, lack of interoperability of equipment, early phase clinical trials, and obtaining FDA approvals.

#### 2.1.3 Need for More Rapid, Effective Sample Preparation

##### Problem

The goal of sample preparation methods is to effectively isolate, extract, and concentrate samples adequate for the microarray analysis. Sample preparation is challenging and is still one of the more time-consuming, labor-intensive, and error-prone steps in the analytical cycle. Specimens are often complex matrices that bind or mask target analytes and contain interfering substances. There is a pressing need to minimize these errors especially with highly parallel assays.

##### Approach

Sampling errors can be minimized by adequately training technicians to use sophisticated biochemical methods and by incorporating consistent automated procedures. Sample integrity can also be validated by the use of appropriate controls including standard templates and test samples. A promising new approach is to implement biological MicroElectroMechanical systems (bioMEMS) for systematic sample preparation and analysis in a highly controlled manner. The bioMEMS-based technique is rapidly evolving and may allow effective sample preparation for the conventional microarray platform. In addition, bioMEMS may lead to a fully integrated single lab-on-a-chip platform including sample preparation, hybridization, and finally the detection.

### 2.2 Flow Cytometry

Flow cytometers measure laser induced fluorescence and light scattering from individual cells passing through a focused laser beam. Immunofluorescence analysis is one of the most common applications. Prior to the flow cytometer measurements, cells are incubated in a solution which contains a selection of antibodies with attached fluorophore labels. The antibodies react with specific antigens on the surface of the cell and the attached fluorophores serve as indicators of the antibodies which are present on the cell surface. A recent trend has been toward simultaneous measurement of multiple fluorophore colors each indicating the presence of a specific antigen on the cell surface. The importance of determining the actual number of antigens on the surface of the cell has driven the need for quantitation in flow cytometry [10].

For most biological applications, cell to cell variability in the quantity being measured using flow cytometry methods is relatively large (> 10 %). These quantities include, but are not limited to, cell volume, cell pigment, membrane fluidity, as well as the amounts of RNA, antigens, and DNA. The exception is the amount of DNA per cell, which in normal, non-replicating cells is constant. Practical measurements of DNA content per cell must have standard uncertainty of 3 % or better. In many other (non-DNA) applications, the amount of a cellular material being measured can vary over a range of three decades or more within a heterogeneous population.

Flow cytometer signals are pulses typically at rates up to 50,000 Hz with durations of 1 to 10 µs, and which produce 0 to 10^7^ photons per pulse. The fundamental measurement is the total (integrated) number of photons (or photoelectrons) in the pulse.

#### 2.2.1 Lack of Linear Measurement Response

##### Problem

Accuracy in flow cytometry measurements is synonymous with linearity or proportionality. A typical immunofluorescence application can require a measurement range of four decades. To provide for the various types of applications an instrument might encounter, an instrument must be capable of measurements over more than six decades. However, linearity over a six decade measurement range is also desirable in a multi-purpose tool. Usually the readout range is limited to four decades by the data acquisition electronics, therefore a portion of the six decade span must be selected. Photomultiplier tubes (PMTs) provide flexible and nearly noise-free gain, and the ability to select the optical (photon) range that will be measured, but PMTs are not perfectly linear detectors over their entire operational range. The four decade range that the data acquisition electronics must cover is also a challenge, addressed either by the use of logarithmic amplifiers or by using linear amplifiers with 14-bit or greater A/D converters.

For a general purpose flow cytometer, accuracy of 1 % or better is desirable. To achieve that accuracy, the nonlinear response must be less than a few percent. The two situations requiring the greatest linearity are measurements of DNA content per cell and correction for spectral overlap of different fluorophores in different detector channels. The stringent requirement for linearity in spectral overlap correction may not be immediately obvious. But in practice, the overlap correction uses linear equations that can involve the subtraction of large numbers. Considering that the data can cover 4 decades, small errors in the large numbers can result in a large absolute error in the subtracted result.

##### Approach

The best method for characterizing linearity has been to use a double pulse method which uses two input signals of constant ratio but varying absolute value to measure deviation from linearity. For a perfectly linear system, the ratio of the measured values will be constant over the entire signal range. Deviation from linearity can be measured relative to an average value. A practical alternative is to analyze a mixture of particles with known relative intensity values and compare the measurement with expected result.

#### 2.2.2 Standards for Quantitation of Fluorescence Intensity and Specific Cellular Material

##### Problem

A fluorescence intensity standard is most useful when its emission spectrum matches a fluorophore used to stain cells. In that case a standard measure of fluorescence production known as molecules of equivalent soluble fluorophore (MESF) can be used. Using a spectrally matched standard and fluorophore of interest insures that spectral variations in optical filters and other optical components do not affect the intensity standardization process. NIST provides a set of fluorescein isothiocyanate (FITC) labeled beads, RM 8640, which is the only nationally recognized fluorescent particle reference material [11]. Several companies provide fluorescent particle intensity standards for other fluorophores, but they are not recognized by any standards setting body.

##### Approach

A possible approach is to develop internally consistent systems for quantitating the amount of antibody bound per cell using highly characterized fluorescent antibodies and spectrally matched particle standards. But this is only widely done for the fluorochrome phycoerythrin. Presently the only reliable standards for quantitating other cellular components use biological standards. An example of this is the cell nuclei from a defined species as a standard for DNA content.

#### 2.2.3 Effect of Instrument Limitations on Particle Fluorescence Measurements

##### Problem

To make precise flow cytometry measurements, one must overcome instrument limitations due to variability at the component level. The sample sizes, ranging from thousands to millions of particles, compound the variability problem. The most common applications require resolving subpopulations of particles based on differences in their average fluorescence intensity. Ideally, the measurement process variability is significantly less than the variability in the sample itself. Since the signal can range over 4 decades or more, measurement uncertainty is limited by different factors (e.g., illumination uniformity, photoelectron statistics, background light, electronic noise) in different regions of signal level. This complicates standardizing or characterizing the uncertainty of measurements.

##### Approach

As a practical way to characterize measurement variability over a wide range of light intensities, a set of particles stained with different amounts of fluorescent dye and with known intrinsic (sample) variances can be analyzed. The increase in measured coefficient of variation (CV) above the known sample CV of each type of particle allows the instrument contribution to CV or variance to be determined at different signal levels. With just a few carefully chosen particle intensities (dim, midlevel and bright on the measurement scale), it is possible to separately determine electronic noise, background light, photoelectron statistics, and illumination uniformity contributions to the measurement variance over the entire 4 decade signal range. Determining intrinsic sample CV within appropriate limits takes a great deal of care and is most difficult for brightly fluorescent particles with intrinsic CV’s less than 1 %.

### 2.3 Optical Microscopy

Optical microscopy has been a staple of biological research since Leeuwenhoek demonstrated its utility in 1674. In contrast to cytometers or microarrays, described above, optical microscopy provides images of biological specimens, ranging from tissue samples, at low magnifications, to images within single cells, at high magnifications. With the advent of multispectral imaging and specific biological labels, more sophisticated optical microscopy techniques are moving beyond the realm of simply providing records of tissue structure and morphology and into the realm of providing quantitative data regarding tissue behavior. These techniques take advantage of the spectral distribution of the optical illumination and how these different spectral components interact with biological tissues and labels. However, even when used to classify tissue samples, and certainly when used to provide enhanced scientific understanding or medical diagnostics, a number of measurement and procedural needs exist, the lack of which can result in image misinterpretation. These needs can conveniently be broken into four categories: hardware, reagents, software, and human involvement.

#### 2.3.1 Hardware

##### 2.3.1.1 Hardware Barriers to Acquiring Accurate Spectral Images

###### Problem

Variations in detector gain [12,13], for example, spectral dependence or nonlinear intensity response, distort images by inaccurately representing analyte concentrations for both single analytes and for comparison between analyte concentrations. In a similar fashion, misaligned optical components reduce sensitivity and resolution, resulting in decreased contrast and broadened analyte locations in images.

###### Approach

Detector and system alignment problems have long been recognized and there are standard procedures to address most of them. Almost all commercial optical instruments are provided with detailed alignment instructions. Routine maintenance, e.g., a weekly alignment check and detector calibration with either a multi-line lamp or multi-wavelength fluorescence standard can minimize alignment and wavelength sensitivity issues. Similarly, detector calibration as a function of intensity, using a calibrated light source [14] can solve the nonlinear detector gain problem. However, difficulties remain with these solutions. Whereas routine calibration procedures will detect temporal degradation of the detector and the optical elements in the light path of the instrument, assuming the presence of stable optical standards, it becomes difficult to maintain the instrumentation if aging of the standards has to be incorporated into the calibration procedure. To counter this problem, it is necessary for the user to have access to traceable standards with a guaranteed lifetime under prescribed storage conditions. Additionally, the standards need to be routinely re-certified [15,16] in a time-frame corresponding to minimum standard degradation. Such timeframes will almost certainly have to be determined empirically, since usage patterns, storage conditions, and environmental parameters will all contribute to degradation rates.

Even with appropriate standards and well defined calibration procedures, there remains at least one further problem in assuring accuracy associated with comparing images obtained on different instruments. Each instrument will have its own limitations. Therefore comparisons between images obtained on different instruments require a thorough understanding of the limitations associated with the specific instruments being used to generate the images.

##### 2.3.1.2 Degradation of Optical Components

###### Problem

The problem of time dependent degradation has been alluded to above. It is important for the user to bear in mind that, in addition to the detector, all optical components degrade with time (filters, lenses, mirrors) due to atmospheric attack on the optical coatings [17]. While this is usually a slow process, occurring over years, it affects image quality and accuracy and, because it is gradual, there is no standard procedure dictating when components should be replaced or recoated.

###### Approach

Temporal degradation of optical components can be monitored through routine, e.g., monthly or bi-monthly, component inspection using a standard source and a calibrated detector.

#### 2.3.2 Reagents and Labels

##### Problem

Probes, such as antibodies, peptides, and nucleic acids, all of which are either naturally occurring or artificially engineered, interact with specific substances within a cell or within tissues [18]. Labeled probes allow detection of specific proteins, chemical reactions, and even time dependent interactions. Consequently, probes have to meet the following conditions: high specificity, high affinity, and appropriate mobility. However, with higher specificity, the number of required probes that are needed to completely assess tissue behavior is greatly increased. Indeed, to obtain more detailed and more specific information regarding tissue response, it is becoming necessary to use multiple probes, each of which exhibits high specificity and high affinity and all of which have mobilities that allow them to concurrently interact with their tissue counterparts.

Labels are the components attached to probes that allow probe tracking and imaging. They can be radioactive, magnetic, or fluorescent, and they can be a natural part of the probe or either a biological or non-biological element attached to the probe. For optical imaging, labels are typically organic [19] or inorganic [20] fluorophores. Regardless of their source, all labels must address the following issues: spectral stability (both intensity and spectral behavior), quenching, toxicity, and mobility [21]. Clearly, the spectral behavior has to be stable; however the environment within tissue is extremely aggressive and can result in chemical attack on the labels destroying or distorting their fluorescence. Fluorescence quenching can occur through multiple paths: photobleaching, proximity to other similar fluorescent sources, or fluorescent resonance energy transfer (FRET). Toxicity is a matter of concern for all *in vivo* and most *in vitro* applications [22]. Currently, there is very little understanding of the possible toxicity issues regarding labels, which are typically sub-micrometer size. However, not only does toxicity increase as particle size decreases, but the toxicity of a particle is also a function of shape, deformability, and stability. As labels increase the overall size of the probes, they exacerbate the mobility issues associated with movement of the probe to reaction sites. Finally, the fluorescent output of labels can be confused with naturally occurring fluorescence from other sources that have no relation to the biological component or reaction of interest.

##### Approach

There is a great deal of ongoing research to address the problems listed above [23]. At this time, no clear, across-the-board solutions have been found. Rather, tradeoffs between the benefits and problems associated with possible solutions must be weighed. For example, probes are being engineered to achieve high specificity and affinity. The ability of the probe to move through the cell or tissue to reaction sites depends upon its size and deformability as well as upon the properties of any attached labels in addition to the specificity already mentioned. However, increased specificity may lead to an increase in the number of probes required to identify suspect tissues and/or cells. There is also the question of toxicity. In general, organic fluorophores, particularly those developed from naturally occurring biological processes, are considered safer than inorganic fluorophores such as quantum dots (QDs), which are alien to biological systems and frequently are composed of toxic elements. However, QDs have far greater brightness and are less prone to photo-bleaching and quenching. Because the optical properties of QDs are controlled by their size, QDs that fluoresce at specific wavelengths can be developed far more rapidly than organic fluorophores can be developed. Understanding of the effect of QDs on probe mobility in biological systems is currently lacking. Unlike organic molecules, QDs cannot deform to allow migration through constricted regions and, because the QD fluorescence wavelength is a function of size, mobility considerations may ultimately restrict the wavelength range for various applications. Nevertheless, QDs overcome some of the most serious problems associated with development of organic fluorophores as labels and are being used both *in vitro* and in small animal studied. Toxicity concerns will prevent their use *in vivo* in human subjects in the immediate future.

#### 2.3.3 Software

##### 2.3.3.1 Linear Unmixing

###### Problem

Linear unmixing is the process by which multispectral contributions to an image pixel are separated into their individual source component contributions. The assumption underlying linear unmixing is that the total signal at any given image pixel is a linear combination [24] of the light signals (i.e., fluorescence sources or chromogen absorbance) plus an error term. The error term includes both the contribution due to noise and uncertainty resulting from imprecise or erroneous fitting criteria. While it is not required that either the number or the shapes of the individual spectra be known *a priori*, tools exist that can infer the source values from the set of image pixels – the uncertainty component varies with the statistical approach used to unmix the data [25] and to a greater extent, with the accuracy and completeness of the spectral endmembers (spectral libraries) used in the unmixing. Such variations alter the quantitative attribution of the image pixel intensities to the individual signals. The unmixing algorithms themselves are well understood and validated, being based on years of non-imaging spectroscopic analytical techniques, but accurate results depend on having optically well-behaved samples (scattering and stray light, for example, can introduce errors) as well as correct spectral endmembers. Methods for creating appropriate spectral libraries vary from the trivial (simply sampling spectra of pure fluorophores or chromogens) to the complex, e.g., multiple curve resolution [26]. As with any involved scientific method, appropriate validation strategies are essential to its successful employment.

###### Approach

However the libraries are generated, the performance of the spectral unmixing method used, namely, the combination of library and algorithm, should be appropriately validated using control samples and/or well-characterized mixtures of labels. The usual standards of the scientific method, if applied properly, should facilitate this process. However, widespread discussion of the details of the mathematics involved (e.g., the use of non-negativity constraints) is probably not necessary; what is more relevant is the performance of conceivably proprietary strategies on publicly available standard samples. Appropriate protocols or guidelines of a general nature that could be used by method developers to work up and test each new combination of sample preparation and unmixing parameters can put these techniques on solid ground. Workshops to address method development and assessment with participation from researchers, image processing software manufacturers, and end users would be useful. Subsequent incorporation of these guidelines into commercial software is essential.

##### 2.3.3.2 Confounding Phenomena

###### Problem

Experimental aspects of confounding phenomena, for example, autofluorescence, noise, photobleaching, quenching, fluorescence resonance energy transfer, etc., have been discussed previously. The potential for interactions between measured signals and sample properties (scattering, absorbance, variable thickness, etc.) also need to be acknowledged. Finally, it is worth mentioning that the entire sample procurement, processing and labeling process is fraught with potential problems. Attempts have been made to address these problems through software development. There are two concerns with this approach: software limitations and operator education. Experimental procedures exist that will identify and, in some cases, compensate for most confounding phenomena. However, data acquisition and analysis software must be flexible enough to allow these procedures and operators must be educated in proper techniques both in recording the data and in subsequent analysis.

###### Approach

One particularly useful approach is to include image diagnostics into the analysis software that could conceivably look for signatures of some of the problems noted above. For example, with linear unmixing, one can monitor the magnitude of the fitting error at every pixel; if it exceeds a certain threshold, that could be an indication that something is wrong either with the sample, the imaging, or the unmixing method. Similar tools could exist to monitor other confounding phenomena. Appropriate correction techniques can be applied. These could include noise filters, intensity scaling capability, image comparison and subtraction tools, and wavelength discrimination capacity. This collection of tools will permit background noise reduction, autofluorescence detection and possible subtraction, and hyper-spectral monitoring (see linear unmixing, above). These tools exist in many image analysis software packages. However, appropriate procedures need to be followed so that inappropriate use of powerful image processing techniques do not distort the data.

A discussion of the Food and Drug Administration (FDA) guidelines for software validity and traceability can be found on the FDA web site [27]. A critical concept emphasized in the document is that software cannot be validated in the absence of instrumentation. All possible hardware and software procedure combinations must be tested before software can be considered validated. In the context of this document, a similar conclusion can be drawn; software must be flexible enough to allow any necessary procedure to identify and minimize the presence of artifacts. Moreover, software documentation should be adequate to provide the user information regarding artifact detection and possible generation as a result of specific image processing steps. However, the user has the responsibility of understanding the implications of image analysis steps, regardless of documentation in the software. Such understanding would derive automatically from standardization of image processing procedures for specific biological applications.

#### 2.3.4 Human Involvement

In the present, and for the foreseeable future, human participation in medical image analysis is unavoidable. This is due, in part, to the fact that medical imaging is undergoing rapid advances that currently preclude complete automation, in part because computer algorithms are not yet as sensitive to optical patterns as the human brain, and, in part because of reluctance for relinquishing control and legal responsibility of such a complicated task to an automated system. However, incorporation of the human mind into image analysis simultaneously incorporates biases that can distort the analysis. It should be noted that solutions to this problem are not independent of the solutions to the hardware data acquisition and software image analysis issues discussed above.

##### 2.3.4.1 If a Pattern is Expected and Observed, It is Assumed to be True

###### Problem

The process of seeing what is expected and, consequently, forgoing critical assessment, is an universal hazard in research activities. This danger is particularly present in image appraisals for two reasons. First, the human mind has evolved to identify patterns, even when obscured by noisy optical backgrounds. The existence of similarly appearing objects in proximity to each other, or with a distribution that evokes a (partially complete) geometric structure, or even in a pattern that appears to contain symmetry elements can lead the mind to interpret patterns that do not exist in the data. Second, images, unlike graphs or charts, cannot contain quantitative uncertainty values conveniently attached to the data. If the image appears to support the expected model, there is a tendency to avoid searching for artifacts or errors.

###### Approach

With the exception of using completely automated image analysis, there is no guaranteed solution to this problem. Training human operators in potential sources and appearances of artifacts will be helpful but not sufficient. In a research environment, peer review and reproducibility requirements are intended to eliminate these types of misinterpretations. However, when the interpretation is based upon partially defined, un-codified criteria, i.e., criteria that are at least partially subjective, peer review is not an efficient filter. In medical applications, wherein missing indications in an image can result in serious health problems and time constraints are important, there is an additional driving force for indication identification without a corresponding balancing criterion for artifact rejection. Automatic image analysis would eliminate the problem of subjective image interpretation. However, for image analysis to be automated, both image preparation (collection procedures and post collection filtering) and image analysis criteria must be standardized and codified. This will require standards developing committees that include users, hardware designers, and software developers. A final caveat is that image analysis software will detect only what has been codified. Therefore, some images, that contain early indications of problems that experienced human analysts might notice, may go undetected by automated procedures.

##### 2.3.4.2 Computer Generated Images are Frequently Accepted as Accurate Although Features of the Image May be the Result of Over Processing

###### Problem

Computer image improvement, for example, filtering, sharpening, contrast enhancement, is based upon mathematical tools and assumptions that are often poorly understood by the user. However, the ease with which these tools can be applied can give rise to over-processed images in which patterns, boundaries, edges, and segregation appear but have no physical significance. Consequently, overprocessed images give rise to faulty conclusions based totally upon artifacts.

###### Approach

There is no completely satisfactory solution to any of the human interpretation issues. The problems can be reduced by a combination of standardization of image analysis procedures and education of the user to the inherent hazards of non-rigorous image interpretation and the creation of artifacts, including meaningless patterns, through image processing and data acquisition.

### 2.4 Optical Coherence Tomography

Optical coherence tomography (OCT) is a three dimensional interferometric biomedical imaging technique capable of *in vivo* measurements. With micrometer-scale spatial resolutions and penetration depths of 1–3 mm in most tissues, OCT fills a useful niche between confocal microscopy, which has high spatial resolution but is difficult to implement *in vivo*, and ultrasound whose spatial resolution is typically 10 to 100 times poorer than OCT. The primary commercial use of OCT at present is in ophthalmic applications for retinal imaging of the human eye. However, a great deal of research and clinical demonstrations of OCT are ongoing in a variety of applications including early diagnosis of arterial plaques, assessment of burn severity, detection of cancer and its precursors, and Doppler measurements of blood flow to name a few.

First demonstrated in 1991 by Fujimoto and collaborators [28], OCT is a relatively new technology with significant promise for growth and new applications. The focus of improvements to OCT has been on increasing measurement speed through improved signal to noise ratios, improving spatial resolution, and developing new measurement modalities such as phase or polarization sensitivity to increase contrast and broaden the diagnostic capabilities. To fully exploit the potential of OCT as a biomedical imaging tool, supporting measurement capabilities (metrology) must be developed simultaneously. There are three significant measurement challenges facing OCT: real time wavelength characterization of OCT tunable laser sources, insufficient data on optical properties of human tissues, and the need for characterization of tissue property changes in response to electromagnetic excitation. Each metrology challenge and proposed technical approaches are summarized below.

#### 2.4.1 Wavelength Characterization of OCT Tunable Laser Sources

##### Problem

The basis of an OCT measurement is to illuminate the specimen with a broad bandwidth of light and coherently resolve the backscattered light to distinguish light returning from different depths in the tissue. To do this, the return light must be resolved either temporally (time-domain) or spectrally (frequency-domain). The time domain approach involves illuminating with a spectrally broad optical source, moving the interferometer’s reference mirror, and recording intensity data as a function of reference arm delay time [29]. The frequency domain approach has a fixed reference arm but resolves the returning light by optical frequency (or wavelength) [30]. This is generally done either with a broadband optical source and spectrometer detection, or with a wavelength-tunable laser source and a detector.

The frequency domain approach using a rapidly tunable laser (swept source) is a promising approach and is receiving attention due to improved noise performance and rapid measurement capability compared with time domain approaches. The signal-to-noise ratio (SNR) of frequency domain approaches can be significantly better than for the time domain with theoretical sensitivity improvements of 20–30 dB [30]. Improved measurement speed of frequency domain approaches comes from this improved sensitivity, which reduces required sampling time, and from the elimination of the need for the relatively slow mechanical motion of the reference mirror in the time domain. Swept-wavelength lasers have demonstrated sweep rates up to 290 kHz (145 nm range) and OCT measurements have been performed with line scan rates of up to 58 kHz [31].

Characterization of fast-swept sources involves dynamic measurements of absolute wavelength, sweep linearity, and instantaneous linewidth. Due to the Fourier transform relationship, the effective depth range goes like π/2δk where δk is the wavenumber spacing between samples of the sweeping laser [30]. This means that poor wavelength resolution can limit the penetration depth of the OCT. Also of practical measurement interest is the limited reliability of current swept wavelength laser sources. Due to the exacting mechanical requirements on most swept sources they tend to drift out of specification in terms of their sweep repeatability and linearity. Therefore, for higher accuracy applications, real time monitoring of laser wavelength during the OCT scan is important.

Optical frequency comb sources also present an attractive possibility as an innovative spectrometry tool for OCT. Optical combs are based on a pulsed laser in a resonant cavity operating in a highly nonlinear regime to generate harmonics. Such sources yield spectra consisting of narrow lines evenly spaced in optical frequency and covering bandwidths of 1 to 2 µm [32]. In both the optical comb source and the swept laser source, it is notable that the instantaneous linewidth of the laser or a single comb tooth is a very small fraction of the total bandwidth range. The spectral range determines the spatial resolution of the measurement and the spectral resolution specifies the maximum depth range. However, since depth range is generally limited in practice by the SNR, swept laser or comb-based OCT sources are capable of providing narrower linewidths than are currently needed. Therefore, it may be advantageous to use these narrow-linewidth sources to spectrally encode information to improve noise averaging or measurement efficiency. This prompts the consideration of source bandwidth efficiency.

##### Approach

Perform accurate characterizations of OCT source wavelength, sweep linearity, and linewidth at video rates. Improved metrology in wavelength characterization (and stability) could allow multiplexing of information onto the same optical bandwidth to improve the information or noise reduction content of the signal in a given optical bandwidth. Technology exists for the real-time monitoring of swept laser linewidth. However it is not widely available and is often too slow to be of use to video rate swept laser OCT systems.

#### 2.4.2 Insufficient Data on Optical Properties of Human Tissues

##### Problem

To fully describe the propagation of light in tissue requires knowledge of the full spatial and spectral dependence of the tissue’s complex refractive index. As a comparison, in the field of non-biological optics, light is refracted and guided through lenses, mirrors, waveguides, etc. A key parameter to describe light’s behavior in any material is the complex refractive index (governing propagation and loss). Extensive data on the refractive indices of optical glasses are published to high accuracy. In contrast, for biophotonic diagnostics, the propagation medium for light is tissue where the ‘database’ of tissue refractive index is sparsely populated. This results in two limitations. First, propagation of light through tissue is more difficult to predict. This is not limited to OCT, but to any instrumentation where light is to be delivered to a particular portion of tissue (including both diagnostic and therapeutic applications). Second, diagnostic techniques ultimately measure local variations in refractive index in order to determine tissue health. Interferometric techniques such as OCT are especially susceptible to small (nanometer scale, in the case of phase sensitive OCT) variations in the optical pathlength experienced by the backscattered photons. A trusted database of human tissue refractive indices will be an important milestone that enables a significant jump in the diagnostic capabilities of OCT and other biophotonic techniques.

The task of characterizing human tissue refractive index is a huge undertaking. Unlike optical-quality glasses, most tissue is highly heterogeneous. For example, in the retinal nerve fiber layer of the human eye, the size scale of various constituents ranges over 3 orders of magnitude with neurotubules (25 nm), mitochondria (1 µm), and cell bodies (10 µm). Tissue also experiences significant anisotropies in its structure resulting in polarization-dependent variations in the complex refractive index such as birefringence and diattenuation.

##### Approach

To create a database of the various constituents of human tissue for refractive index as a function of wavelength. Of course, to be useful, this data would have to be combined with an understanding of the relationship between tissue properties and measured refractive index. In other words, the measurement uncertainty will depend on the sample-to-sample variability of refractive index. This will involve some characterization of the tissue with regard to its health. Finally, the characterization of tissue index will need to be applied to modeling to yield predictions of the coherent properties of light as it is backscattered from the various tissue types. Because of the complexity of the problem, a panel of experts should rank order the priority for acquiring the information to populate this database.

#### 2.4.3 The Need for Characterization of Tissue Property Changes in Response to Electromagnetic Excitation

##### Problem

The dielectric susceptibility of a medium describes the intrinsic properties affecting electromagnetic propagation through that medium. This includes the previous discussion of the effect of the complex refractive index on propagating light. On the other side of this interaction is the effect that electromagnetic fields have on the tissue as they propagate. This property of the tissue’s “electromagnetic response” is important to both the understanding of the health and function of the tissue as well as to determine how light will propagate during a photonic diagnostic or therapeutic procedure.

One example is in the electromagnetic excitation (action potential) of neural tissue (axons). An electromagnetic pulse from the brain instigates physical changes in the nerve including motion of the axon which is detectable through phase-sensitive OCT. Development and characterization of OCT-based measurement strategies to probe tissue electromagnetic response will be important to fundamental understanding of tissue function and its related pathology.

Characterization of light propagation in tissue modified by its “electromagnetic response” is particularly important for understanding tissue response to laser stimulation. For example, laser-induced heating of tissue can produce deformations of the tissue (thermo-elastic effects) and changes in the refractive index (thermo-refractive effects). Both of these effects will modify the propagation character of the applied laser light depending on laser wavelength and power. Understanding these properties is necessary to properly characterize the propagation of light during photonic diagnostics (to invert measurement results to yield tissue properties) or during therapeutic applications (to direct and localize the applied light).

##### Approach

Develop measurement techniques for assessing nanometer scale response of tissue to electromagnetic stimuli. Characterize the propagation parameters of tissue in response to the applied fields of the light source

## 3. Summary

This manuscript focuses on four major areas of clinical applications of biophotonics at both the macroscopic and microscopic scales: microarray technology for assays of DNA and proteins, flow cytometry technology for measurements of antigens on the surface of a cell, optical imaging for *in vitro* diagnostics of pathological tissue conditions, and optical coherence tomography for *in vivo* imaging and diagnostics. There are other optical probing methodologies, such as laser-induced stimulation and vibrational spectroscopy, under development and it is likely that some of them will find applications in the clinic. However, the purpose of this paper is to focus on the critical measurement needs for tissue and cell diagnostics. The field of biophotonics is developing rapidly and its symbiotic relation with nanotechnology through improved artificial markers such as quantum dots and nanocrystals, is expected to have increasing impact on the quality of health care. Progress will depend on identification of technological obstacles and the development of solutions to these obstacles.
